# “Please speak in Hindi”: Cognitive interviews to improve measurement of digital access in North India

**DOI:** 10.1371/journal.pone.0354874

**Published:** 2026-07-28

**Authors:** Zenia Taluja, Anjora Sarangi, Pankaj Mishra, Chandrika Prasad Verma, Manjula Sharma, Osama Ummer, Arjun Khanna, Sara Chamberlain, Priyadarshini Krishnaraj, Megha S. Hudda, Amnesty E. LeFevre, Kerry Scott

**Affiliations:** 1 Devsol Research Consultant Private Limited, New Delhi, Delhi, India; 2 Johns Hopkins India Private Limited, Pune, Maharashtra, India; 3 School of Public Health and Family Medicine, University of Cape Town, Western Cape, South Africa; 4 Centre of Social Medicine and Community Health, Jawaharlal Nehru University, New Delhi, India; 5 2X Digital, New Delhi, India; 6 School of Global Health, Faculty of Health, York University, Toronto, Canada; 7 Department of International Health, Johns Hopkins Bloomberg School of Public Health, Baltimore, Maryland, United States of America; PLOS: Public Library of Science, UNITED KINGDOM OF GREAT BRITAIN AND NORTHERN IRELAND

## Abstract

Access to and use of mobile phones are increasingly a prerequisite for full participation in modern society. Yet, a significant digital gender gap persists in India, where women are less likely than men to own phones or access the internet. To measure and understand this gap, survey questions must be both comprehensible to the respondents and contextually appropriate. We conducted 101 cognitive interviews with men (n = 44) and women (n = 57) in rural Uttar Pradesh to test 118 draft survey questions developed for an upcoming survey on digital access and use. These draft questions were drawn primarily from validated global instruments, with some additional questions newly developed by our team, and were all translated into Hindi. Through team debriefs and systematic analysis of the interview transcripts, we identified seven categories of question problems: (1) inappropriate terminology, (2) overly complex wording, (3) low resonance of digital concepts (e.g., personal data, online tracking, privacy policies, hacking), (4) confusion when asking about permission and supervision of mobile phone use due to phone sharing and overlap with concept of help or support, (5) problematic question structures and response options (especially Likert scales), (6) self-practice bias, and (7) unclear time frames and recall expectations. Overall, we found that many draft survey questions were incomprehensible to respondents despite our research team’s efforts to develop clear and simple translations. Revisions were required for almost all questions. Our findings highlight the critical role of cognitive interviewing in improving data quality, particularly when conducting surveys across languages and sociocultural contexts, and amid rapidly changing digital landscapes.

## Introduction

Mobile phone access has been rising steadily across the world, enabling relatively cheap and effective communication, as well as access to information and vital services on health, education, society, and the economy [1]. The role played by mobile phones became particularly prominent during the COVID-19 pandemic, highlighting the importance of phone connectivity in accessing essential services and staying connected [2]. Access to mobile phones is positively associated with multiple indicators linked to global socioeconomic development [3] including health service use and empowerment [4–6]. Mobile phones have become a vehicle to implement a number of development efforts, especially in low- and middle-income countries (LMICs), such as health information campaigns, targeted financial services, online tutoring, voter registration, and public benefits or entitlements.

The Mobile Gender Gap Report 2023 [1], published by GSMA, finds that despite growing phone use, men have higher rates of mobile phone ownership and access in many settings. The report highlights that across LMICs, women are 7% less likely than men to own a mobile phone, with the mobile gender gap being widest in South Asia at 15%. India account for nearly half of the world’s gendered digital divide, with 81% of men compared to only 72% of women owning mobile phones. Beyond ownership, women’s use of smartphones and access to the internet on mobile phones is also lower, a gender gap that is particularly significant because phones are the primary way most people in LMICs access the internet. Further, the report notes that the gendered digital divide is more pronounced in rural areas owing to socioeconomic factors such as limited capacity to afford phones, lower literacy rates, and more restrictive social norms for women. This gender gap is both a manifestation of historical inequity and a driver of current and future inequity. With lower access to and use of phones, women continue to lose out on the wide range of economic, social and health benefits of mobile phones.

The ability to monitor trends in phone ownership and use is vital for understanding the gender gap and for designing effective digital programs. Despite the need for this data, significant gaps and inconsistencies remain in how digital access and adoption are measured. Many national surveys have space for only one or two questions on these topics (such as whether the respondent has a phone or uses the internet), leaving policymakers without information on issues ranging from digital skills to normative beliefs about phone use. Surveys often showcase divergent conceptualizations of the digital constructs being measured. For instance, phone ownership has been framed as owning a personal phone, having a phone that you can use, owning an active SIM card, or possessing a phone that is in working condition [7]. In addition, some survey questions are not aligned with LMIC contexts. For example, UNICEF’s Multiple Indicator Cluster Survey-6 (MICS-6) focused on computer use, despite the fact that the vast majority of digital technology use in LMICs occurs via mobile phones not computers. (MICS-7 has broadened to include mobile phones.) Finally, questions on digital access and use may be developed and implemented without sufficient testing and adaptation to local languages and contexts, potentially resulting in survey questions that are incomprehensible or irrelevant for some populations. Given these gaps, a broader library of robustly designed questions is needed to better capture the breadth of digital skills, access, and use.

In order to develop this set of questions and support an upcoming survey in India, our team undertook cognitive interviewing to test–and improve– the relevance and comprehensibility of survey questions on digital access and use. This study reports on the cognitive interview findings from Uttar Pradesh, a Hindi speaking state of India. The tested questions will be considered for a population level survey on digital access and use in the same region. Additional cognitive interviewing is planned for Kenya (Kiswahili) and Nigeria (Hausa) to further develop the library of questions for use in other surveys.

## Methodology

### Cognitive interviewing

Cognitive interviewing can be defined as ‘the administration of draft survey questions while collecting additional verbal information about the survey responses, aimed at evaluating the quality of the response or helping determine whether the question is generating the information that its author intends.’ [8] This research methodology identifies and remedies mismatch between respondent interpretation of survey questions and researcher intent. The process of cognitive interviewing generates insight into the linguistic quality of translations [9–11] and the cultural and context-specific resonance of words and phrases in questions [12]. As a result, the implementation of cognitive interviewing extends beyond determining whether respondents interpret questions as intended to examining the constructs captured by the survey questions and whether those constructs are understood consistently across culturally diverse respondents [13].

This study followed the methodological approach detailed in Scott, Ummer & LeFevre [14]. During cognitive interviews, our researchers asked survey questions exactly as written, as if administering a quantitative survey, and attempted to elicit a response based on the quantitative response options available. Then, the researchers asked a series of qualitative probes to assess how the respondents interpreted the questions, why they gave the responses provided, and whether their interpretation of the question matched the research intention. Testing each question took between one and five minutes: less than a minute to administer the quantitative question and then several minutes to discuss the question with the respondent in order to assess the question’s comprehensibility before moving on to the next question [14]. Extensive debriefs, central to the cognitive interviewing methodology, were held at the end of each interview day. During these debriefs, the research team discussed each question and the responses elicited from research participants. When misunderstandings were identified the researchers revised the questions. The revised questions were taken to the field for further testing with new respondents. After three rounds of testing and revision, the survey questions were finalized for use in quantitative large-scale surveys [14].

### Developing survey questions for testing and cognitive interview guides

After reviewing and mapping questions on digital access and use from major surveys, we identified priority questions for cognitive testing. We also added new questions proposed by our team to fill measurement gaps for a total of 118 questions to be tested (these questions, both the initial version tested and the final version developed, in Hindi and English, are available in S1 Appendix). These questions sought to measure key outcomes of interest across the domains of (a) phone ownership, access, and use; (b) differences in how men and women use the phone and the internet, (c) norms and attitudes about phone and internet use, and (d) concerns and harms related to the phone and internet use.

During a week-long intensive workshop, our research team undertook a rigourous translation process. Each question was read and discussed at length in English until all researchers understood its intended meaning (i.e., the construct it was seeking to assess), then multiple Hindi translations were developed, compared, and discussed. The researchers drew from their experience working in rural Uttar Pradesh to propose words and phrases that they thought would be most comprehensible and straightforward for the respondent population. Back translations were developed and examined. Further discussion and adjustments to the Hindi translation resulted in a final draft version of all 118 questions.

These 118 questions were divided across three cognitive interview guides, i.e., approximately 40 questions per guide, so that each cognitive interview took no more than an hour and a half. Each quantitative survey question to be tested was followed by probes to assess understanding, such as “Why did you say [response provided]?”; “Could you tell me in your own words what this question is asking?”; “What does [keyword] mean to you?” and “Is there any other word for [keyword] that people generally use here?”. Researchers also asked tailored probes (also called adaptive follow up questions) based on the respondent’s specific replies to uncover individual insights, which would be overlooked by fixed protocols and survey questions [14,15]. For instance, if a respondent earlier stated that she did not know what the internet was, but later said she learned a new recipe by watching YouTube, the researcher asked a follow up question to assess her understanding of the word “internet” and to explore what alternative words or examples are available to better convey the concept of “internet.”

### Study location and participant sampling

We selected adults with fewer years of formal education than average, living in more remote geographic regions of Uttar Pradesh, whom we approached under the guidance of local community leaders. It was assumed that educated urban residents would be more likely to find the draft questions comprehensible. Testing the questions with adults who had limited formal education in rural areas would reveal the weaknesses of the questions, thereby enabling revisions that improve comprehensibility for the entire target population.

We cognitively tested the questions in rural areas of Badaun district in the west and Jaunpur in the east of the state. In both districts, Hindi is the primary language of communication and is the language used for major surveys (such as the Indian National Family Health Surveys). Also, we selected respondents who had used a mobile phone at least once in the last two weeks. Since many survey questions focused on respondents’ phone use, someone who had not used a phone at all over the last two weeks would skip most questions, rendering the interview ineffective for testing the comprehensibility of those questions. As we proceeded through the study, we also increased focus on smartphone users because several of the survey questions that we wanted to test were linked to internet use and would otherwise be skipped for those with basic phones.

**Fig 1:**
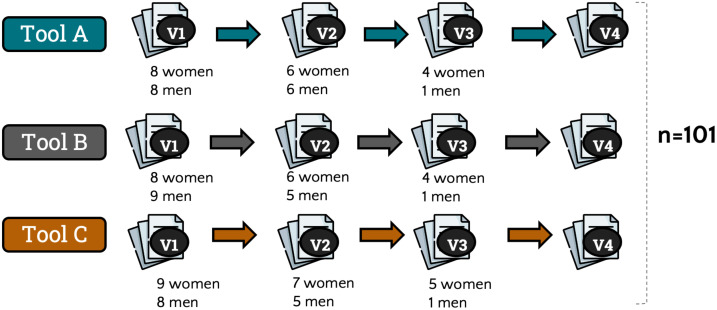
Iterative sampling approach for cognitive interviews in Uttar Pradesh, India.

Guided by the concept of thematic saturation [16], we interviewed a total of 101 different respondents across three rounds (Fig 1) between 01/04/2023 and 27/04/2023. The questions were revised for each subsequent round to produce version 2, version 3 and then version 4, which was the final version and was not tested. We required fewer respondents per round as the questions became increasingly comprehensible [14]. We also found that male respondents found the questions easier to understand, therefore additional female respondents were sampled to ensure the questions were comprehensible for all.

### Ethics statement

Ethical considerations are essential in research to protect participants’ rights, welfare, and dignity, while maintaining scientific integrity, accountability, and societal trust [17]. Accordingly, this study adhered to established ethical standards and received ethical approval from the Sigma Institutional Review Board (IRB) (IRB Number: 10123/IRB/22–23) in India and the Johns Hopkins Bloomberg School of Public Health IRB (23938) in the United States. Only interested and eligible respondents were approached by the qualitative researchers, and interviews were set up at a time most suitable for the respondents. Additional information regarding the ethical, cultural, and scientific considerations specific to inclusivity in global research is included in the Supporting Information (S1 File).

### Data collection

The cognitive interviews were conducted by five female (ZT, AS, MS, PK & MSH) and two male (PM & CPV) qualitative researchers. All had master’s level social science education. Their experience as qualitative researchers ranged from five to 25 years; selecting experienced qualitative researchers was considered particularly important for cognitive interviewing, as effective tailored probing relies on researchers’ interviewing experience and skills, ensuring methodological rigor, ethical sensitivity, and credible interpretation during interviews [14]. The researchers were from the study region (Uttar Pradesh) or had worked there previously. A logistics manager initiated the process of recruitment by approaching the village head (sarpanch), explaining the study, and taking help from the sarpanch or a community health worker in identifying potential respondents.

Interviews were conducted at respondent’s homes in teams of two where one researcher was responsible for leading the interaction and the other was accountable for notetaking and supporting the interaction. The study information and informed consent was read to each potential participant and then summarized to ensure comprehension. All respondents provided informed oral consent to participate in the study and have the conversation recorded, apart from one woman who refused for the conversation to be audio recorded. Interviews averaged 52 minutes in duration.

### Data analysis

Analysis began during fieldwork at our extensive team debrief sessions [14]. For each day of data collection (every eight to 12 interviews), the team engaged in a full day of debriefing to discuss emergent cognitive mismatches and identify potential revisions. During these sessions, language and translations were refined, question order was revised to improve flow, response options were amended, alternative questions were developed, and questions that did not resonate with the respondents’ context were eliminated. While debrief sessions were aimed at refining the tool until cognitive interviews demonstrated better comprehension of the survey questions amongst respondents, these sessions also helped inductively gauge a preliminary understanding of the types of recurring cognitive mismatches on the basis of which revisions to the tool were also made. After data collection, audio recordings of the 70 most substantive interviews were translated in English with key Hindi terms retained in the transcripts. The different types of cognitive mismatches identified through team discussions and debrief sessions were used to code interviews on a qualitative analysis software (Dedoose). The remaining 31 interviews that were not transcribed were analyzed through thorough reading of the extensive notes maintained in the debrief sheet.

### Findings

Respondents were rural men (n = 44) and women (n = 57), who ranged in age from 18 to 47 years (average 30 years). Respondent characteristics are presented below (Table 1). Only 20% of the men in our sample and 18% of the women had nine or more years of education, which is lower than the Uttar Pradesh rural average of 81% of men and 62% of women [18]. All the men and 81% of the women in our sample reported owning their own phones, while the remaining 11% used someone else’s phone or shared a household phone. This phone ownership status was higher than the Uttar Pradesh average: NFHS-5 [19] found that 94% of rural households possessed at least one phone (no data specific to men) and 42% of rural women had their own phones. 84% of our male respondents and 56% of our female respondents owned smartphones. While there is no comparable data on smartphone ownership, our sample likely had higher smartphone ownership than the Uttar Pradesh average: a representative 2022 survey of households with school-going children in Uttar Pradesh found that 68% of households had a smartphone [19].

**Table 1:** Sample population (N = 101).

Characteristic	Male (N = 44)	Female (N = 57)	Total (N = 101)
Own a phone	44 (100%)	46 (81%)	90/101 (89%)
Do not own a phone	0 (0%)	11 (19%)	11/101 (11%)
Own a smartphone	37/44 (84%)	32/57 (56%)	69/101 (68%)
Own a feature phone	2/44 (5%)	4/57 (7%)	6/101 (6%)
Own a basic phone	5/44 (11%)	10/57 (18%)	15/101 (15%)
Age: Average in years (range)	31 (18–47)	29 (19–45)	30 (18–47)
Education: Average in years (range)	7.3 (0–10)	6.9 (0–14)	7.0 (0–14)
0 years	1/44 (2%)	1/57 (2%)	2/101 (2%)
1–5 years	10/44 (23%)	15/57 (26%)	25/101 (25%)
6–8 years	25/44 (57%)	32/57 (56%)	57/101 (56%)
9+	9/44 (20%)	10/57 (18%)	19/101 (19%)

Nearly half the respondents belonged to the “Other Backwards Caste” category (a somewhat marginalized group), almost 40% were in the highly marginalized Scheduled Caste/Scheduled Tribe category and the remaining 13% were in the “General” category (not marginalized). About a third of the respondents reported their annual household income when asked, which ranged from Rs 35,000 (USD$427) to Rs 11,00,000 (USD$13,414), with an average of Rs 1,82,000 (USD $2219) across families ranging in size from 4 to 13 people. Although a number of dialects of Hindi are also spoken across Uttar Pradesh, as well as the distinct languages Urdu and Bhojpuri, all potential respondents whom our researchers approached, and all respondents included in our study were Hindi speakers and completely comfortable conversing in Hindi.

Drawing on previous studies [12] concerning the typology of survey question failures, we identified seven question issues that affected comprehension (Table 2). Each of the seven issues from Table 2 are elaborated upon in the sub-sections below.

**Table 2:** Typology of problems identified in the digital survey questions.

Question issue	Explanation	Example
1. Inappropriate terminology	Vocabulary words and formal terms were not locally understood or were interpreted in unanticipated ways.	• The initial translations for keywords such as chance/likelihood, unmarried, inappropriate, offensive, and health worker were not understood. • Using the phone for financial transactions (translated to “give/take money”) was understood by some men to mean using the phone to access a loan.
2. Long and overly complex questions	Wordy questions with multiple qualifiers were incomprehensible to many respondents; In some cases, respondents retained only part of questions, resulting in an interpretation that did not align with question’s intent.	• When asked “Do you personally own a mobile phone? Please do not include old handsets that you do not use anymore”, some respondents replied “no” but explained that they have a phone however it is a new phone.
3. Low resonance of digital concepts	Some key digital concepts were not locally understood, leading to confusion and mismatched interpretation.	• While many respondents used WhatsApp and YouTube, they did not classify these activities as using the internet. • Privacy policy, personal data and hacking were all unfamiliar concepts that were difficult to explain in a meaningful way.
4. Difficulties in conveying the concepts of ‘permission’ and ‘supervision’	Conveying the social control aspect of “needing permission to use a phone” and “being supervised when using a phone” was tricky and highly context**-**specific	• Needing permission to use the phone or being supervised while using the phone was often understood by women as asking for assistance or receiving help. • In contexts where respondents shared phones with other family members, asking before using the phone was considered more of a courtesy to inform others than truly requiring someone’s permission. • Being overseen or supervised when using a phone may be linked to simply having others around (in a shared living space) while using the phone.
5. Problematic question construction and response options	While adding a sentence to transition between sections of the survey worked well, question preambles that contained directions for respondents and question stems were not retained. Replying to questions by indicating one’s degree of opinion or intensity of agreement did not resonate. Questions that repeated the same structure but with subtly different components were considered to be asking the same thing multiple times.	• The following preamble was not retained: “I now want to understand whether there are any reasons/causes due to which you face trouble using the net on a phone. Even if you do not use the net on a phone, or only use it sometimes, I would still like to understand more.” • A question stem of “In the last seven days have you…” was forgotten as we proceeded through the question sub-parts. • When asked whether the internet’s effect on the respondent’s life was “very positive”, “somewhat positive”, somewhat negative”, or “very negative”, respondents converted the response options from a four point Likert scale above to simply “positive” or “negative”.
6. Self-practice bias	Respondents interpreted questions on what others think to be asking what they themselves do. Even when understanding the question’s focus on others’ attitudes, respondents struggled to speculate.	• When asked whether one’s family members agreed that women could learn new things to earn money through using the internet on their phones, female respondents explained that they had or had not done this. • When then asked whether the family or community agrees, many women said that they could not know what others think.
7. Unclear time frames and recall expectations	It was unclear that frequent behavior fit within a broader umbrella time period (“within the last three months”); reporting typical behavior was difficult.	• Respondents did not interpret having used the internet yesterday as falling within the umbrella concept of “having done so within the last three months”. • When asked about typical or average use, respondents had responses like, “when I need the phone, I use it, when I don’t need it, I leave it.”

1. Inappropriate terminology

Although the researchers sought to select commonly used Hindi words for the first draft of the questions, many words selected were unfamiliar to our study population, especially those with lower education levels. One interviewee (F27), when asked a question about being exposed to “inappropriate or offensive things” [*anuchit ya aaptijanak cheeze*] on the internet, summarized this issue by replying: “Please speak in Hindi. What does the question mean?” In fact, the questions were entirely in Hindi but used more formal Hindi words that were not familiar to respondents. In most cases, we were able to identify a more appropriate word or phrasing (Table 3) that is commonly used in everyday language to improve comprehension among respondents of differing educational backgrounds.

**Table 3:** Vocabulary issues and potential resolutions.

Challenge	Original	Improved alternative word/ phrase identified
Original word not locally understood	Sambhavana [possibility/likelihood]	Ho saktaa hai [it is possible]
	Aavivaahit [unmarried]	Kunwara [bachelor (masc)] / Kunwari [virgin (fem)]
	Vois kaal [voice call]	Aadiyo kaal [audio call]
	Vois sarch [voice search]	Bol kar khojna [searching by speaking]
	Anuchit ya aapattijanak cheezein [inappropriate or offensive things]	Galat cheez [wrong things]
	Nigraani [supervision]	Nazar rakhna [watch/keep and eye on]
Original word or phrase has unanticipated connotations that distorted the meaning of the question	Paise ka len-den [give-take money (lit); money transactions]; understood by some to mean taking a loan	Paise bhejte yaa mangwaate [send or receive money]
	Kaam dhandhe [work business]; understood by some to mean domestic work	Paise kamaane, kaam dhoondhne, samaan bechne ya thekedaar se baat karne [Earn money, find work, sell goods or talk to contractors]
	Gaane [songs]; understood to mean entertaining music but not religious devotional songs	Gaane jaise bhajan, qaawali, ya filmi gaane [songs like bhajans (Hindu devotionals), qawwalis (devotional/sufi) or songs from films]
	Dost [friend]; understood to mean only male friends	Dost / saheli [friend (masc) / friend (fem)]

While many words were completely unfamiliar to respondents, others were understood but interpreted in a manner consistent with their context but misaligned with the researchers’ intent. For instance, one question asked whether respondents had used the phone for work or business [kaam dhandhe]. While the question intention was focused on using phones for income**-**generating activity, some female respondents understood the question to be asking about domestic work.

Researcher: *So, as I asked, since yesterday you have used a mobile for work or business. What does “kaam- dhandha” [business] mean to you?*

*Respondent: “Kaam-dhandha” means cooking food, washing utensils. All the household chores. (F06)*

Furthermore, female respondents reported never speaking to or having friends when we used the term *dost* [friend]. Upon probing, we learned that *dost* was gendered as a male friend in this region. The term for female friend, *saheli*, allowed women to consider whether they spoke to female friends.

2. Long and overly complex questions

Many questions were too long and complex, causing respondents to lose track of the question’s intent. For instance, we tested the following question about SIM use, drawn from a global survey: “You said earlier that you don’t own or have the main use of a mobile phone. However, do you own or have the main use of any SIM cards (i.e., mobile phone numbers) that you use in other people’s mobile phones at least once a month?” Most respondents were not able to meaningfully retain and process the five clauses (owning a SIM or having the main use of a SIM; a SIM is a mobile phone number, using one’s SIM in another person’s mobile, doing so at least once a month). In several cases, respondents lost the intent of complex questions completely, replying “I don’t know.” In some cases, however, respondents retained and addressed only part of questions, which did not align with question’s intent (Table 4).

**Table 4:** Examples of overly long and complex questions that led to partial retention.

Issue	Original complex question	Respondent interpretation and sample response	Revised simplified question
The inclusion of an example introduced too much information; respondent focused only on the example	Since yesterday, have you used your mobile phone to speak with your relatives? Like speaking with your mother, father, brother-sister, mother-in-law or father-in-law on WhatsApp. (Source: new)	Respondent understood the question to be whether they use WhatsApp to speak to family members: “*No. […] I haven’t used WhatsApp… I don’t do much on WhatsApp. I usually make calls to talk to my family members*.” (F03)	Since yesterday, have you used a mobile phone to talk to relatives?
	Since yesterday, have you used your mobile phone to talk with friends? Like messaging or speaking to your friends on WhatsApp? (Source: new)	Respondent focuses on whether she uses messaging functions: ***Respondent****: No. […] I do not know how to message. (F08)* ***Researcher****: But have you talked to friends?* ***Respondent****: Yes (F08)*	Since yesterday, have you used a mobile phone to talk to friends?
Respondent focuses on only one clause	Do you personally own a mobile phone? Please do not include old handsets that you do not use anymore. (Source: GSMA Consumer Survey 2022)	Respondent focuses on whether she uses old handsets. ***Respondent****: No.* ***Researcher****: What did you understand from the question?* ***Respondent****: You asked me if I used my old mobile phone. (F04)*	Do you personally own a mobile phone?
	Have you ever used the internet from any location on any phone or computer? (Source: adapted from MICS-7 original: “Have you ever used the internet from any location and any device?”)	Respondent focuses on the use of the internet on a computer only: “*No. […] I haven’t used the internet on a computer*.” (F11)	Have you ever used the net?
Respondent focuses on the indefinite determiner or adverb	Have you used the internet on any phone? (Source: new)	Respondent interprets use on “any phone” to mean “any other person’s phone” ***Respondent****: No, I haven’t used it.* ***Researcher****: Why did you say no, didi [sister]?* ***Respondent****: Because I don’t take the net from anybody else, because the net is already there in my mobile. So why should I ask [for the net] from anyone else?* ***Researcher****: If I ask you, if you have used the net on your mobile phone, what would you say?* ***Respondent****: Yes (F28)*	Have you ever used the internet on a mobile phone?
	On balance, has using the internet on a mobile phone had a positive or negative impact overall on your life in the last 12 months? (Source: GSMA Global Consumer Survey 2022)	Respondent focuses on “on balance” [translated as “overall” in Hindi] rather than “your life” and interpreted the question to be asking about broad societal effects.	Has using a mobile phone had a positive or negative impact on your life?

Questions were more accessible when we removed clauses and simplified the wording. Unfortunately, these simplified questions lost some specificity. For instance, a simplified question only asked about “talking” to friends without specifying that this could be text messaging or speaking. In some cases, separate follow up questions would be required to access all areas of interest.

3. Low resonance of digital concepts

Many concepts related to digital services were unfamiliar to respondents. In some cases, as with ‘the internet’ and ‘apps’, respondents were engaging with these technological tools but did not link their practices with the broader concept. For instance, majority of the respondents had not heard of the internet (or ‘net,’ a more common but not universal term in the area) but were in fact using WhatsApp regularly. Respondents did not know the term ‘app,’ but knew how to press the red icon on the phone to open YouTube. We were able to resolve this issue by introducing an explainer box that informed respondents that using specific digital services and programs was using the internet and apps. The explainer box was successful when it mentioned specific well-known products or companies such as YouTube, WhatsApp, Google, Meesho, Moj, Jio Cinema, Flipkart, and PayTM.

Other digital concepts, such as privacy policies, personal data, and hacking, were simply not resonant among the respondent population. Each of these terms brought unique challenges. We tested survey questions that sought to assess whether respondents read and understood privacy policies. However, the term privacy policy had no accessible direct Hindi translation. Attempts to describe a privacy policy involved long, complex sentences that were incomprehensible to some respondents. Among the respondents who followed, our attempted explanation mapped onto a different but related concept: the steps required to create user accounts rather than privacy policies.

***Researcher*** *[reading the draft question]: Many times, when we download a new app or go to a new website, before using it for the first time, we need to read some kind of notice [privacy policy] and give our permission. Have you ever seen something like this?*

***Respondent*** *: Yes, while making an ID they ask for name and OTP [one-time-password]. (M20)*

We tested questions that sought to assess whether respondents knew about data protection and companies collecting their personal data. To do so, we first established whether respondents understood ‘personal data’. The best translation was *niji jaankaari* [personal information]. However, this term has interpretations unrelated to data, such as family secrets or day to day practices in the home.

***Researcher** [reading the draft question]: Have you ever heard of personal information?*

***Respondent**: Yes. Personal information means household, food, cooking, what has happened to whom in the family, who is going where, all that. Children’s studies. (F49)*

Asking about online companies such as Facebook and WhatsApp collecting personal information added several layers of complexity. In addition to ambiguity around the concept of personal information, respondents understood companies to be physical factories rather than providers of online services, and conflated collection of personal data with how public or private the online space was.

***Researcher*** *: According to you, do companies or various apps like Facebook and WhatsApp collect your personal information?*

***Respondent*** *: Facebook is not your personal life. That is known to all. When you are using Facebook, another person who is using Facebook, that person will know your number and identity. That person will know that this Facebook account is run by this person. That is not personal. But WhatsApp is my personal app. (F40)*

We tested a question that sought to assess whether respondents’ phones or accounts had ever been hacked. The English word “hack” has entered global usage but had no relevance to our respondent population. Instead, it was instead consistently misheard as “hang,” another English term used in Hindi to mean a system freeze or slowdown.

***Respondent*** *: My phone was hacked but it got recovered.*

***Respondent*** *: Like touch screen is not working or phone is not working.[…] if mobile’s storage is full then it gets hacked. (F23)*

Attempting to describe the concept of hacking as someone accessing or controlling your phone from a geographically distant location did not resonate. Only one respondent (M38) understood this concept. For this and other digital concepts (privacy policy, personal data), we used explainer boxes, but they were met with limited success.

4. Difficulties conveying permission and supervision

Questions about permission and supervision sought to gauge agency and surveillance over the use of digital devices. We asked respondents whether they needed permission to use the phone or were supervised when using the phone for different activities, and whether they thought these conditions should apply to women, men, young unmarried women, and young unmarried men in general. What we observed was a conceptual blurring between the idea of social control inherent to the English words permission and supervision, and the more benign concepts of notification (“I am taking the phone, I want to talk, tell me where the phone is kept.” F03) and assistance (“Educated people will use it on their own, others will ask” F08). The trickiness associated with these concepts was also context**-**driven where, despite owning a phone, both men and women reported sharing their phones with other family members when required, and it was thus common courtesy to ask for permission when using someone else’s phone or to inform others about using a shared phone.

***Researcher*** *: Do you think women should take someone’s permission (anumati lena) to use a mobile phone?*

***Respondent*** *: Yes, they should take permission because if they don’t know how to use it, then they can ask. (F06)*

***Researcher*** *: Ok then tell me, do you think women should use the internet on a mobile phone without having anyone watch or supervise them (bina kisi ki dekh rekh ke)?*

***Respondent*** *: You can do as per your wish, but when someone will supervise you, then you can understand things easily. (F04)*

After testing the questions, we observed that the control aspect of taking permission could only be assessed in relation to the respondent’s phone ownership status. Specifically, if a phone owner is required to seek permission before using their own phone, that would indicate control, as would having to always ask permission whenever one wants to use a shared phone. The control aspect of supervision continued to be tricky particularly because the actual Hindi word for supervision, *nigrani*, was not understood by respondents, the term *nazar rakhna* (keep an eye on) approximated the meaning better than *dekh rekh* (watch over).

5. Problematic question construction and response options

While preamble text that simply helped respondents’ transition to a new section was not problematic, preamble text that provided important guidance to respondents was not retained. Relatedly, questions built around a “stem” and then several sub-components were unsuccessful because respondents did not retain the stem once they moved into the sub-components.

For questions which were worded similarly but had differences in key clauses, some respondents found it difficult to distinguish the questions and considered them to be the same question being repeated. For example, questions about communicating with strangers and communicating with men outside the home were understood to be asking the same thing, despite the research intent to differentiate the gender-agnostic “strangers” from the gender-specific “men” (Table 5).

**Table 5:** Example of similar questions that respondents considered to be identical.

Questions	Intended difference	Respondent perspective
QA. Women face harm at home if they communicate on the internet with people whom their husbands and families don’t know. Does this happen around here? (Source: new) QB. Women face harm at home if they communicate on the internet with men who do not live in their households. Does this happen around here? (Source: new)	Question A was about communicating with strangers while B was about communicating with men (known or unknown)	*Both questions are the same.* (F18) *It is the same thing.* (M36)

Questions that sought responses along a binary or a three-point Likert scale (good/neutral/bad; agree/neutral/disagree; often/rarely/never) were successful. However, longer Likert scales were less effective as the idea of having degrees of agreement or disagreement was unfamiliar to respondents, resulting in confusion for both respondent and the interviewer in categorizing the response.

6. Self-practice bias

We sought to assess respondents’ perceptions of the attitudes held by their families and communities. Typically, our questions included a statement followed by a question such as “what do your family members think about this?” or “what do people in your community think about this?” We observed two major challenges; first, when questions were asked in a quantitative format the first time, respondents often reported their own practice or opinions. Either they did not understand that the question was seeking family/community members’ opinions or they conceptualized their experience as indicative of their family members’ views. Second, when probed further by researchers, some respondents still found it challenging to report others’ opinions and attitudes. Many respondents would either say that they didn’t know what their family members thought or that they were unwilling to speak on their behalf. Some had never had conversations about views on phone use and so were unable to arrive at an estimation of others’ opinions and attitudes.

***Researcher*** *: Women who use the internet on a mobile phone can earn more money. What do your family members think about this?*

***Respondent*** *: I don’t earn money. Rather, I buy all the necessary things. (F15)*

*No, I don’t know so much about people. […] It does not happen in my family. (F42)*

***Researcher*** *: Does this happen when women post photos or videos of themselves or others on Facebook, WhatsApp, YouTube or Instagram?*

***Respondent*** *: I don’t do that madam. (F44)*

Respondents indicated that their families’ views on these issues could be discerned only in relation to what was actually happening. That is, if women in the home were taking specific actions--using the phone to learn new things, saving time and effort through using the phone, helping their child children with schoolwork through using the phone--then the family was supportive. If women were not using the phone for family oriented and productive work, then it was difficult to know families’ perspectives on this potential action. Respondents also found it very unclear what “community” was supposed to mean, with some thinking of their caste community and others noting that they could not possibly know what everyone in the community thought. The best data was captured from reported personal practice rather than questions on opinions about others. However, questions can ask respondents what they think and what their families think to capture the difference between the two cognitive domains.

7. Unclear time frames and recall expectations

We identified three issues with time frames and recall. First, most respondents struggled to report activity within pre-set categories. When asked whether they had done an activity within the last one month or within the last three months, respondents did not conceptualize frequent use as falling within these umbrella categories. Phone use was a frequent activity for many of the respondents, which made it difficult to report frequency of phone use behavior over a longer time period. For example, when asked whether they used a SIM card in their phone within the last one month, respondents who did so every day were unsure how to reply. A few respondents interpreted the question to be asking whether they had engaged in the particular activity one month ago or three months ago, rather than *within* one or three months. Some respondents also reported that thinking back over the course of a month or three months was too long a recall period. We found success with questions that asked whether the respondent had ever performed an activity, and, if yes, when they last did the activity.

The second challenge was with regard to aggregating and estimating time spent on activities. Respondents were unable to articulate what an ‘average’, ’typical’, ‘overall’ time period in a day was for a mobile phone-related activity. Instead, they sometimes narrated incidents related to the activity. Distilling multiple separate activities into a concrete set of hours and minutes was challenging for respondents.

*If I get to use it, I will. If I don’t, if I’m busy, I can’t. If the phone is at home, I use it. And if I don’t, it’s not necessary. […] If I get a call it stays in my hands. If I am working or am busy, I keep it on a shelf or in the room or kitchen. If I go to someone’s house, I carry my phone with me. (F14)*

*I don’t use it every day. Only whenever one gets time. (F11)*

*It depends on the time of the day, and how long I talk. (F08)*

When this question was edited to include the day or time of day the activity was carried out – for instance, ‘yesterday’, ‘whole day’, ‘mostly in the mornings’ ‘mostly in the evenings’ etc., it improved comprehension among respondents.

The third challenge was that the Hindi word for ‘week’ [*saptah* or *hafta*] was not consistently understood by respondents. A respondent said a week is made up of eight days, while another said two weeks constitute a month. Days were understood so rather than asking about a week, we found that asking about seven days was better understood. We also noted that respondents discussed time in relation to events, such as harvests and religious festivals. However, anchoring time**-**related recall questions in a survey would be difficult.

## Discussion

Cognitive interviews conducted in rural Uttar Pradesh revealed substantial cognitive gaps between the intended meaning of survey question and how respondents interpreted them. Many challenges we identified—particularly those related to translation, question length, and response formats—have been reported in research with other marginalized populations [20–22], including earlier work on measuring respectful maternity care in Hindi-speaking north or central India [12]. However, our findings also highlight several underexplored issues specific to measuring digital access and use in LMIC settings.

*Language, translation, and question complexity*: Several comprehension problems stemmed from the interaction between translation choices and question complexity. Although Hindi equivalents existed for most English terms, many were highly formal or technical and not used in everyday speech. Respondents often did not recognize these terms, prompting requests that interviewers “please speak Hindi,” despite the questions already being translated into standard Hindi. When equivalent colloquial terms are unavailable, researchers often rely on longer explanations to convey technical concepts. This creates a trade-off between clarity and brevity: longer questions increase cognitive load [23], and can lead respondents to answer based on only part of the question. Breaking complex questions into shorter items may improve comprehension, but it can also lengthen surveys and increase respondent fatigue [24–26]. Our findings underscore the importance of prioritizing natural, commonly used language when translating survey instruments and carefully balancing clarity with respondent burden [27,28].

*Challenges measuring digital access and use*: Several issues related specifically to digital measurement mirrored findings from prior research by organizations such as BBC Media Action [29] and the GSMA [30]. Respondents often used locally meaningful terminology—for example, referring to smartphones as “touch phones”—and found time references framed in days easier to understand than those framed in weeks. Cognitive interviews also highlighted complexities in measuring phone access in contexts where devices are frequently shared and where permission or supervision may shape use. These findings reinforce the value of cognitive testing when adapting digital access measures for rural or resource-constrained contexts.

*Measuring attitudes and perceived social norms*: Eliciting quantitative data on attitudes and perceived community views proved particularly challenging. Many respondents struggled with conventional four- or five-point Likert scales, which are commonly used when measuring attitudes [31,32], echoing findings from earlier research in rural India [12]. Respondents also had difficulty answering hypothetical questions about what others might think and instead responded that they do not know what others think or responded by describing their own behavior, a pattern we term self-practice bias. This bias may reflect the strength of social norms. For example, when asked whether her family would approve of a woman using social media, a respondent would simply report that she herself uses social media—perhaps because her behavior implicitly indicates family approval, since engaging in a disapproved behavior would be unlikely. Simplifying response formats and limiting hypothetical scenarios improved comprehension. In our study, binary responses or three-point scales (e.g., fully agree, somewhat agree/disagree, fully disagree) were more intuitive for respondents and may offer a more appropriate approach when measuring attitudes in similar contexts.

*Cultural framing of time and experience*: Cognitive interviews also revealed differences in how respondents conceptualized time. Standard survey recall periods such as “in the past two weeks” did not always align with how participants remembered events. Instead, respondents often recalled phone use in relation to specific needs or occasions. Such event-based recall patterns have been observed in other cultural contexts [33,34] and suggest that time-based questions may require adaptation when used in rural or low-income settings.

*Implications and novelty*: Overall, our findings demonstrate how cognitive interviewing can uncover mismatches between survey design and respondent interpretation that may otherwise compromise data quality. While previous research has documented challenges with translation, question complexity, and Likert scales in low-resource settings, our study extends this work by identifying cognitive gaps specific to measuring digital access and use. In particular, the “self-practice bias” we observed when respondents were asked to report perceived attitudes within their family or community has not, to our knowledge, been documented previously.

These insights highlight the importance of iterative cognitive testing when developing structured survey tools on digital access in rural contexts. By identifying locally meaningful terminology, appropriate response formats, and culturally resonant ways of framing questions, cognitive interviewing can help produce survey instruments that more accurately capture patterns of digital access and use among marginalized populations.

### Challenges and limitations

Our sample over-represented phone owners and smartphone owners, particularly female smartphone owners. This sampling decision enabled us to collect feedback on a wider range of questions (rather than having to skip some questions due to irrelevance). However, it also skewed our sample towards women who are privileged in their access to phones and therefore, are likely more familiar with technical terminology related to phone use. To address this limitation, researchers conducting cognitive interviews would ask respondents whether certain terms were widely used in the village or whether alternative terms would be more commonly used. The researchers also discussed key issues (such as data privacy, whether phone use should be supervised, and whether people should ask permission before using the phone) with a wide range of respondents, not just smartphone owners.

We deliberately excluded more educated and urban people, reasoning that revising questions to be comprehensible to a less**-**educated rural population would also produce questions that were easily understood by more**-**educated urban respondents. However, this exclusion means we lacked insights from those with higher education and who live in urban areas. We are thus cautious about dropping questions on complex digital topics, such as personal data and hacking, since people outside our sampling population may have greater awareness of and opinions on these topics.

We noted that cognitive interviews are taxing for respondents. Unlike conversational interviews, they flow less naturally and ask respondents to explain thought processes and unfamiliar words or ideas, sometimes leading to frustration or anxiety about being tested. We mitigated these relational challenges by building rapport, clarifying our goals, expressing empathy when questions were confusing or repetitive, and emphasizing the value of their input.

Privacy during the interviews was another challenge. The respondents’ houses, where we conducted the interviews, often had limited private space and were occupied by family members who were curious about the research. Younger female respondents were often checked on throughout the interview by mothers-in-law, elder sisters-in-law or husbands. While we aimed for privacy, we did not insist on it to avoid compromising women’s safety. Instead, we explained the interview’s purpose to family members and politely requested space. Sensitive questions were postponed or skipped when privacy could not be ensured.

Finally, some of our questions on technology-facilitated harms or gender norms risked introducing new fears or problematic ideas. Asking whether women should be supervised or raising topics like hacking sometimes planted concerns that respondents had not previously considered. A few respondents said that our questions were introducing new reasons to worry or even to limit women’s phone use. While our research team was able to counter these views during a post-interview conversation with the respondents, such framing risks influencing public opinion if deployed at scale and must be carefully managed. Despite these challenges and limitations, cognitive interviews provided critical insights into question comprehension among rural Hindi-speaking respondents, enabling targeted refinements that enhance instrument accessibility and validity.

## Conclusion

Cognitive interviews revealed major problems with the questions in a mobile phone access and use survey developed from existing global surveys and expert input. These problems would not have been identified through typical pilot testing and would have undermined the survey’s validity. Addressing these issues required identifying locally appropriate Hindi words and time recall periods, explaining technical terms, re-framing concepts (including permission and supervision in relation to phone use) to be contextually relevant, focusing more on practice and personal opinion than the perceived opinions of others, and greatly simplifying our questions.

Our findings have broader implications. Reliable gender-disaggregated data on digital access remains limited, constraining evidence-based policymaking and monitoring of progress toward gender equity, including commitments under the Sustainable Development Goals and the priorities highlighted during the March 2023 session of the Commission on the Status of Women [35]. Without such data, it is difficult to assess progress toward gender equity, including within the Sustainable Development Goals. Since data is a critical driver of policymaking, the reliability and relevance of data collection methods require greater attention, especially as digital inequalities, including the gender gap, are shaped by diverse contextual challenges. Our results suggest that surveys adapted from global instruments may perform poorly in rural or marginalized contexts unless they are carefully adapted to local linguistic and cultural realities. For instance, the gap between formal standardized Hindi and colloquial everyday language illustrates how technically correct translations can still fail to convey intended meanings. Therefore, questionnaires designed to assess these issues must be carefully crafted to account for wording, translation, and the cultural relevance of specific questions. Cognitive interviewing plays a vital role in this process by uncovering flaws in questionnaire design that often go unnoticed during standard pilot testing.

Future research should continue testing digital access measures across diverse linguistic and cultural settings, particularly in contexts where phones are shared, digital terminology is evolving, and social norms shape technology use. Integrating cognitive interviewing into standard survey development can improve the accuracy and equity of data collection across diverse cultural and linguistic settings, including the rapidly evolving field of digital access.

## Supporting information

S1 Appendix Complete set of all questions tested in cognitive interviews. (XLSX)

S1 File Inclusivity in global research. (DOCX)
